# Charitable Giving for HIV and AIDS: Results from a Canadian National Survey

**DOI:** 10.1371/journal.pone.0103184

**Published:** 2014-08-25

**Authors:** Dan Allman, Liviana Calzavara, Catherine Worthington, Mark Tyndall, Alix Adrien, Melissa Walters, Samantha White, Marcella K. Jones

**Affiliations:** 1 Dalla Lana School of Public Health, University of Toronto, Toronto, Ontario, Canada; 2 School of Public Health and Social Policy, University of Victoria, Victoria, British Columbia, Canada; 3 Department of Medicine, University of Ottawa, Ottawa, Ontario, Canada; 4 Faculty of Medicine, McGill University, Montreal, Québec, Canada; 5 Faculty of Medicine, University of Toronto, Toronto, Ontario, Canada; UNAIDS, Trinidad and Tobago

## Abstract

**Background:**

For the first time, a national survey of adults in Canada posed questions on charitable giving for HIV and AIDS. The objective of this analysis was to explore the behaviour and attitudes of this population in terms of charitable giving.

**Methods:**

In 2011, individuals in Canada 16 years of age or older were recruited for a survey from an online panel supplemented by random digit dial telephone interviewing. The margin of error was +/−2.1 percentage points (95%). Chi-square tests were used to detect bivariate associations. A multivariate logistic regression model was fit to compare those who had donated to HIV and AIDS in the past 12 months with those who had donated to other disease or illness charities.

**Results:**

2,139 participated. 82.5% had donated to a charitable cause in the past 12 months. 22.2% had ever donated to HIV and AIDS, with 7.8% doing so in the past 12 months. Individuals who had donated to HIV and AIDS versus other disease or illness charities tended to be younger (p<0.05), single (p<0.005), more highly educated (p<0.001) and to self-identify as a member of a sexual minority group (p<0.001). Multivariate analysis revealed individuals who self-identified as a member of a sexual minority group were significantly much more likely to have donated to HIV and AIDS than to other disease or illness charities in the past 12 months (OR, 7.73; p<0.001; CI 4.32–13.88).

**Discussion:**

Despite a generally philanthropic orientation, relatively few respondents had ever been involved in charitable giving for HIV and AIDS. Those who had could be understood relationally as individuals at closer social proximity to HIV and AIDS such as members of sexual minority groups.

## Introduction

For more than three decades, Public Health practitioners in Canada have tracked the incidence, prevalence and factors associated with the Human Immunodeficiency Virus (HIV) and Acquired Immune Deficiency Syndrome (AIDS). During this time, researchers, non-governmental and governmental organizations, health care and service providers, community stakeholders and others have worked together to generate knowledge and to develop and deliver an array of programs and services designed to raise public awareness. In the absence of a cure, the main focus of Canada's Public Health response remains the prevention of the transmission of HIV, the reduction of stigma and discrimination, and the increase of the length and quality of life of people living with HIV and AIDS (PLHIV).

Despite these efforts, in Canada, the number of new HIV infections continues to increase. With advances in treatment, and declines in HIV-related mortality, the number of PLHIV also is increasing. Canada's HIV epidemic is concentrated rather than generalized. That is, among specific sub-populations (for example, gay, bisexual and other men who have sex with men, injecting drug users, Aboriginal Peoples, and people who immigrated from countries where HIV is endemic), the prevalence of HIV is substantial enough and concentrated enough that it leads to continuing HIV transmission and infection within these sub-populations. (In contrast, a generalized epidemic is one where HIV-positive people are evenly distributed across populations, risk groups and social strata, and as such, maintains a generalized distribution of HIV infections). Given that Canada's HIV epidemic is concentrated, research and prevention efforts tend to strategically target sub-populations deemed most vulnerable to HIV infection, rather than the general public [1].

A blueprint document for Canada's response to HIV recognized the importance of tracking and understanding the public's view of HIV to ensure that ongoing government support as well as private and corporate donations for HIV continue and/or are increased [2]. In line with this blueprint, over the past 30 years, a number of general population surveys have been conducted to gain a better understanding of Canadians' behaviours, attitudes, knowledge, and perceptions of HIV and AIDS. The first national general population survey was conducted in 1989 by Michael Ornstein [3], and in the years prior to the study reported in this paper, two other general population surveys were commissioned by the Federal Government of Canada. These were conducted in 2003 and 2006 by EKOS Research Associates Inc., a survey market research firm [4]–[5]. A comparison of the results from the 2003 and 2006 national population surveys identified a number of significant changes, both positive and negative, in Canadians' knowledge about HIV, where they were accessing information, and levels of HIV stigma [5].

In 2011 university researchers conducted a survey of the general public with regard to issues relevant to HIV. The survey included questions related to knowledge and awareness of HIV and other infections, knowledge about specific issues related to HIV and AIDS including modes of infection, perceptions on seriousness and curability, treatments, attitudes towards people living with HIV and AIDS, level of comfort around HIV and AIDS, knowledge of people who had died of AIDS, availability and frequency of HIV testing, information-seeking behaviour, criminal prosecution of HIV non-disclosure, perceived effectiveness of condoms, number of primary and casual sexual partners and charitable giving for HIV and AIDS [6]. The 2011 survey updated some of the information provided by the previous national studies. Additionally, and for the first time, the survey explored the behaviour and attitudes of the Canadian population in terms of charitable giving for HIV and AIDS. This is the focus of the present analysis.

### Charitable Giving and Altruism

While a substantial literature exists on charitable giving [7]–[9], very little has considered charitable giving specifically for HIV and AIDS [10]. The 2007 Canada Survey of Giving, Volunteering and Participating found that Canadians donated a total of 10 billion dollars to causes, with religious organizations being the biggest beneficiaries, receiving 46% of the total dollar value of donations [11]. Other recipient classes include health organizations (15%), social services organizations (9%), international organizations (6%) and hospitals (6%). The 2007 study found that most Canadians (84%) aged 15 and older reported making financial donations to charitable or non-profit organizations, with 42% of the total value of donations being collected in places of worship, followed by 16% being donated in response to requests through mail. Tax credits are a factor; 46% of donors responding to the 2007 study indicated that they or someone in their household would claim an income tax credit for any donation made over the past 12 months, with those who give the largest amounts most likely to do so. The prospect of better tax credits as a motivator, however, does not appear to increase with the amount given [11].

While the majority of Canadians made financial donations, a minority accounted for most of the dollars donated. The top 25% of donors (those who contribute $364 or more annually) accounted for 82% of the total value of donations. Those who gave the most were more likely than others to be older, report higher household income, more formal education, to be married or widowed, and to be religiously active. Although donors with higher household income tended to donate larger amounts, those with lower income tended to donate more as a percentage of total income [11]. A 2010 review of the literature on charitable giving identified eight mechanisms as the most important motivators and facilitators of charitable giving: these included awareness of need, solicitation, costs and benefits, altruism, reputation, psychological benefits, values and efficacy [12].

Eveland & Crutchfield (2007) describe how in the early years of the HIV epidemic, funding for HIV and AIDS service organizations tended to be informal, personal, and centred around the social and political networks of those most affected [10]. Once established foundations became involved in HIV and AIDS grant-making, their influence leveraged further support and helped to begin to change the public's perception concerning PLHIV and the needs of HIV prevention, care, treatment and support. Yet, stigma and discrimination persist, and the role of *othering* arguably continues to impact on altruism and charitable giving for HIV and AIDS [13].

The aim of this analysis is to better understand the characteristics of people in Canada who donate to Public Health causes like HIV and AIDS.

## Materials and Methods

### Ethics Statement

The study received ethics approval from the University of Toronto Research Ethics Board.

### Methods

The survey was conducted in Canada's two official languages, English and French, and administered in all of Canada's provinces and territories. Individuals who were 16 years of age or older and living in Canada at the time of data collection were eligible to participate. All respondents were recruited via random digit dialing and surveyed using a mixed-mode methodology for data collection. This consisted of telephone and online panel components. The mode of interviewing employed for any given respondent was based upon their choice as well as the availability of respondents to panel interviewers. At the core of the mixed-methodology process was a sampling frame based on probability theory where all elements of the target population had an equal chance of being selected into the sample. At the first stage of recruitment, a sample was randomly generated from a dual landline-cell phone sample frame. The sample was loaded into an Interactive Voice Response system (IVR). The IVR system made an initial call and up to three repeat calls before numbers were retired from the sample. The IVR surveying incorporated a recorded script questionnaire which allowed for consistency across individuals. Respondents provided their answers to the recorded questions by pressing the appropriate buttons on their telephone keypad and the system recorded their responses into a survey database. Once respondents had completed the general questions, they were asked if they would like to participate in additional surveys with the panel. If so, they moved to the next stage of the recruitment process: live interviewer confirmation. At this second stage of the recruitment process, the interviewer introduced the respondent to the panel's compensation scheme, recorded basic demographic data (gender, age, postal code) and provided the respondent with a choice to participate by telephone or over the Internet. Email addresses were confirmed in real time during the conversation for those indicating a preference for online survey completion.

The survey contained questions regarding demographics and a range of HIV knowledge, attitudes and behaviours. Specific to this analysis were questions exploring respondents' patterns of past charitable giving to causes including HIV and AIDS, intentions for future charitable giving, attitudes towards private sector philanthropic activities and the role of governments in funding and continuing to fund HIV and AIDS research. Questions about respondents' charitable giving history were asked only of those who indicated that they had donated to charity in the past 12 months. Given the sensitive nature of the survey and inclusion of questions not previously asked, the study was pre-tested among a sample of 100 respondents.

Data were analyzed with Stata/IC 13.1. Following best practices as outlined by Public Works and Government Services Canada [14], a non-response bias analysis was conducted on the survey data whereby three variables from the survey sample (age, gender, and province/territory of residence) were compared to equivalent parameters of the general population 16 years of age and older. A descriptive analysis was conducted for socio-demographic characteristics. To examine bivariate statistics, given the weighted survey design, chi-square tests were used and coefficients converted to F-values using a Rao-Scott correction [15]. A multivariate logistic regression was utilized to detect significant (p<0.05) associations with the binary outcome (those who donated to HIV and AIDS versus those who donated to other disease or illness charities) after controlling for potential confounding variables. Unless stated otherwise, all results are weighted using an interlocking weight to balance age, region and gender so that the sample is proportionate to the Canadian population. Missing responses for individual questions were coded as such and not included in our analysis. The survey was conducted by the Strategic Council, a Canadian market research firm with extensive polling experience.

## Results

2,139 respondents completed the survey. Overall, the un-weighted sample closely reflected the actual distribution of Canadians in terms of key variables such as gender, age and province/territory (as per the Canadian Census 2011) [16]–[17] (Table 1). A total of 1,682 (79%) respondents were recruited and participated online and 457 (21%) were recruited and participated by telephone. Of the participants who responded by telephone, 86% responded via a land line telephone and 14% responded by cell phone. The response rate for those who were recruited online was 18% versus 31% for those who were recruited over the telephone. The blended response rate to the survey was 26%. Descriptions of the socio-demographic characteristics of the sample along with variation by mode of data collection are contained in Table 2.

**Table 1:** Key variables from the general population in Canada, 16 years of age and older, compared to the un-weighted and weighted survey samples (n = 2139).

Variable	Canadian Population (%)^a^	Survey Sample (Un-weighted, %)	Survey Sample (Weighted, %)
**Age Group**			
16–24	14.4	12.4	14.8
25–39	23.7	26.0	24.3
40–59	36.4	35.4	34.9
60+	25.5	26.2	26.1
**Gender**			
Males	48.5	49.4	48.4
Females	51.5	50.6	51.6
**Region**			
British Columbia	13.4	12.9	12.3
Alberta	10.6	10.1	10.2
Saskatchewan	3.0	3.7	3.0
Manitoba	3.5	3.9	3.5
Ontario	38.3	36.7	38.2
Quebec	23.9	23.8	24.3
New Brunswick	2.3	2.0	2.4
Nova Scotia	2.8	4.0	3.1
Prince Edward Island	0.4	0.4	0.3
Newfoundland and Labrador	1.6	1.3	1.6
Yukon Territory	0.1	0.8	0.8
Northwest Territories	0.1	0.4	0.4
Nunavut	0.1	0.1	0.1

a Source: Data from Statistics Canada, Census of Canada 2011, Profile of Canada, provinces, territories (cumulative).

**Table 2:** Socio-demographic Characteristics and Mode of Survey Completion, Overall Sample (n = 2139, Weighted %).

	Mode of Survey Completion			
Variable	Online	Telephone	Total	p-value^a^
**Mode** (n = 2139)				
Online	-	-	78.6	-
Telephone	-	-	21.4	
**Age** (n = 2139)				
16–24	13.2	20.5	14.8	<0.001
25–39	28.0	10.6	24.3	
40–59	35.7	31.9	34.9	
60+	23.1	37.0	26.1	
**Gender** (n = 2139)				
Male	49.3	45.0	48.4	0.115
Female	50.7	55.0	51.6	
**Education** (n = 2123)				
≤High School	14.2	54.8	22.9	<0.001
Some/All College	32.6	25.5	31.1	
Some/All University	35.4	15.1	31.0	
Graduate/Prof. Degree	17.8	4.6	15.0	
**Household Income** b (n = 1781)				
<$40,000	22.9	47.2	27.6	<0.001
$40,000–$80,000	33,0	35.1	33.4	
$80,000+	44.2	17.7	39.0	
**Marital Status** (n = 2079)				
Single	36.1	55.3	40.2	<0.001
Married	63.9	44.7	59.8	
**Sexual Minority** (n = 2092)				
No	94.5	98.3	95.3	<0.001
Yes	5.5	1.7	4.7	

a Pearson Chi-Squared, corrected for survey design and converted to F-statistic.

b Annual Pre-Tax.

In our study, the mode of survey-administration (online or telephone) was contingent upon a respondent's choice and the ability of panel interviewers to access respondents at a given time and location. Telephone became the mode employed to survey harder-to-reach individuals within the randomly recruited panel. This resulted in demographic variation by mode. Compared to the online self-administered mode, those who completed by telephone were more likely to be women, single, heterosexual (i.e., non-sexual minority), over age 60, earn less than $40,000, and report high school education or less.

### Sociodemographic Characteristics and Charitable Giving

83% of survey respondents reported that they had donated to any charity in the past 12 months. Women were marginally more likely to donate compared to men (84% versus 81%). The proportion donating in the past 12 months tended to increase with age, education, being employed in the past 12 months, greater reported household income, and reported religious affiliation (data not shown).

### Types of Charities to which Donations Have Been Made

Of those who had donated in the past 12 months, respondents were most likely to donate to organizations that were seeking a cure or treatment for specific diseases and illnesses (62%). Substantial proportions also donated to charities assisting children and youth (51%), disaster relief organizations (42%) and hospitals (32%). When those who had made a donation to a disease or illness charity in the past 12 months were probed on the specific disease or illness, a small cluster of charities tended to be the beneficiaries of these donations. Among this cluster, cancer-based charities were almost universal among donors (96%). Additionally, 45% identified heart and stroke as the disease or illness to which they had donated in the past 12 months, 21% had donated to multiple sclerosis (MS), and 20% to diabetes (data not shown).

### Donating to HIV and AIDS Causes

Of the respondents who had donated to any disease or illness charity in the past 12 months, 22% reported having donated to HIV and AIDS. Bivariate analysis shows that among these individuals, ever having donated to HIV and AIDS was significantly associated with age, education, household earning, marital status, and self-identifying as a sexual minority (Table 3).

**Table 3:** Bivariate Socio-Demographic Associations with Ever Donating to HIV and AIDS among Participants who Donated to HIV and AIDS in the past 12 Months (n = 1765, Weighted %).

	Ever donated to HIV and AIDS				
Variable	Yes	No	Don't Know	Total	p-value^a^
**Age** (n = 1765)					
16–24	10.8	13.3	8.4	12.0	0.024
25–39	24.0	22.7	22.9	23.0	
40–59	38.7	33.6	43.8	36.3	
60+	26.5	30.4	24.9	28.7	
**Gender** (n = 1765)					
Male	42.2	48.5	49.2	47.2	0.078
Female	57.8	51.5	50.8	52.8	
**Education** (n = 1752)					
≤High School	16.9	23.5	14.2	20.6	<0.001
Some/All College	30.6	29.8	38.0	31.2	
Some/All University	31.1	32.2	31.3	31.9	
Graduate/Prof. Degree	21.4	14.5	16.5	16.3	
**Household Income** b (n = 1472)					
<$40,000	23.5	24.8	15.4	23.1	0.002
$40–$80,000	35.4	35.8	30.6	34.9	
>$80,000	41.2	39.4	54.0	42.0	
**Marital Status** (n = 1721)					
Single	39.1	39.4	27.8	37.6	0.003
Married	60.9	60.7	72.2	62.4	
**Sexual Minority** (n = 1726)					
No	89.1	96.7	97.6	95.1	<0.001
Yes	11.0	3.3	2.4	4.9	

a Pearson Chi-Squared, corrected for survey design and converted to F-statistic.

b Annual Pre-Tax.

Of those who had donated to a disease or illness charity in the past 12 months, individuals more likely to have ever donated to HIV and AIDS included women (58%) versus men (42%); those who were 40–59 years of age (39%) or over 60 years of age (27%) compared to those 25–39 years of age (24%) or those between 16–24 years of age (11%); those with some college or a college degree (31%) or some university or a university degree (31%) compared to those with a graduate or professional degree (21%), or those with less than a high school education (17%). In addition, individuals who described themselves as belonging to a sexual minority were more likely to report donating to HIV in the past year (11% of sexual minority respondents versus 3% of other respondents).

While 22% of respondents reported ever having donated to HIV and AIDS, 8% of respondents indicated donating to HIV and AIDS in the past 12 months. Individuals who had donated to HIV and AIDS versus other disease or illness charities in the past 12 months were more likely to be younger, have less education, lower household income, be single and be a member of a sexual minority (Table 4). Comparatively, donors to disease or illness charities other than HIV were more likely to be older, married and report higher household income.

**Table 4:** Socio-Demographic Associations with Participants who Donated to HIV and AIDS versus Other Disease or Illness Charities in the Past 12 Months (n = 1412, Weighted %).

	Donation to Disease and Illness Charities in the Past 12 Months			
Variable	Donated to HIV and AIDS	Donated to Other Diseases and Illnesses	Total	p-value^a^
**Age** (n = 1412)				
16–24	18.2	7.9	8.9	<0.001
25–39	26.1	20.6	21.2	
40–59	30.2	39.8	38.9	
60+	25.6	31.7	31.1	
**Gender** (n = 1412)				
Male	49.8	45.5	45.9	0.334
Female	50.2	54.5	54.1	
**Education** (n = 1402)				
≤High School	28.4	21.0	21.8	0.041
Some/All College	28.7	32.5	32.1	
Some/All University	22.6	31.1	30.2	
Graduate/Prof. Degree	20.4	15.5	15.9	
**Household Income** b (n = 1175)				
<$40,000	30.5	19.9	21.0	0.026
$40–$80,000	29.1	35.1	34.5	
>$80,000	40.4	45.0	44.6	
**Marital Status** (n = 1379)				
Single	49.2	33.7	35.2	<0.001
Married	50.8	66.3	64.8	
**Sexual Minority** (n = 1384)				
No	82.3	97.5	96.0	<0.001
Yes	17.7	2.5	4.0	

a Pearson Chi-Squared, corrected for survey design and converted to F-statistic.

b Annual Pre-Tax.

A multivariate logistic regression model was fit to compare those who had donated to HIV and AIDS with those who had donated to any other disease or illness in the past 12 months (Table 5). The final model displayed includes only those variables that were statistically significant (p<0.05). Controlling for age, individuals who self-identified as a member of a sexual minority were greater than seven times more likely to have donated to HIV and AIDS than those who did not self-identify as a sexual minority (OR, 7.73; p<0.001, CI 4.32–13.88).

**Table 5:** Multivariate Logistic Regression Model for Donating to HIV and AIDS versus Other Disease or Illness Charities, among Participants who Donated to Disease and Illness Charities in the past 12 months (n = 1384, Weighted).

Variable	Odds Ratio	Std. Err.	[95% Conf. Interval]		p-value
**Age**					
16–24	1.00	–	–	–	–
25–39	0.54	0.17	0.29	1.00	0.049
40–59	0.36	0.11	0.19	0.65	0.001
60+	0.42	0.13	0.22	0.78	0.006
**Sexual Minority**					
No	1.00	–	–	–	–
Yes	7.73	2.31	4.31	13.88	<0.001

Note: Modelled using Hosmer-Lemeshow's Goodness-of-Fit test.

### Role of Public Sector and Private Sector in Funding

**Figure 1:**
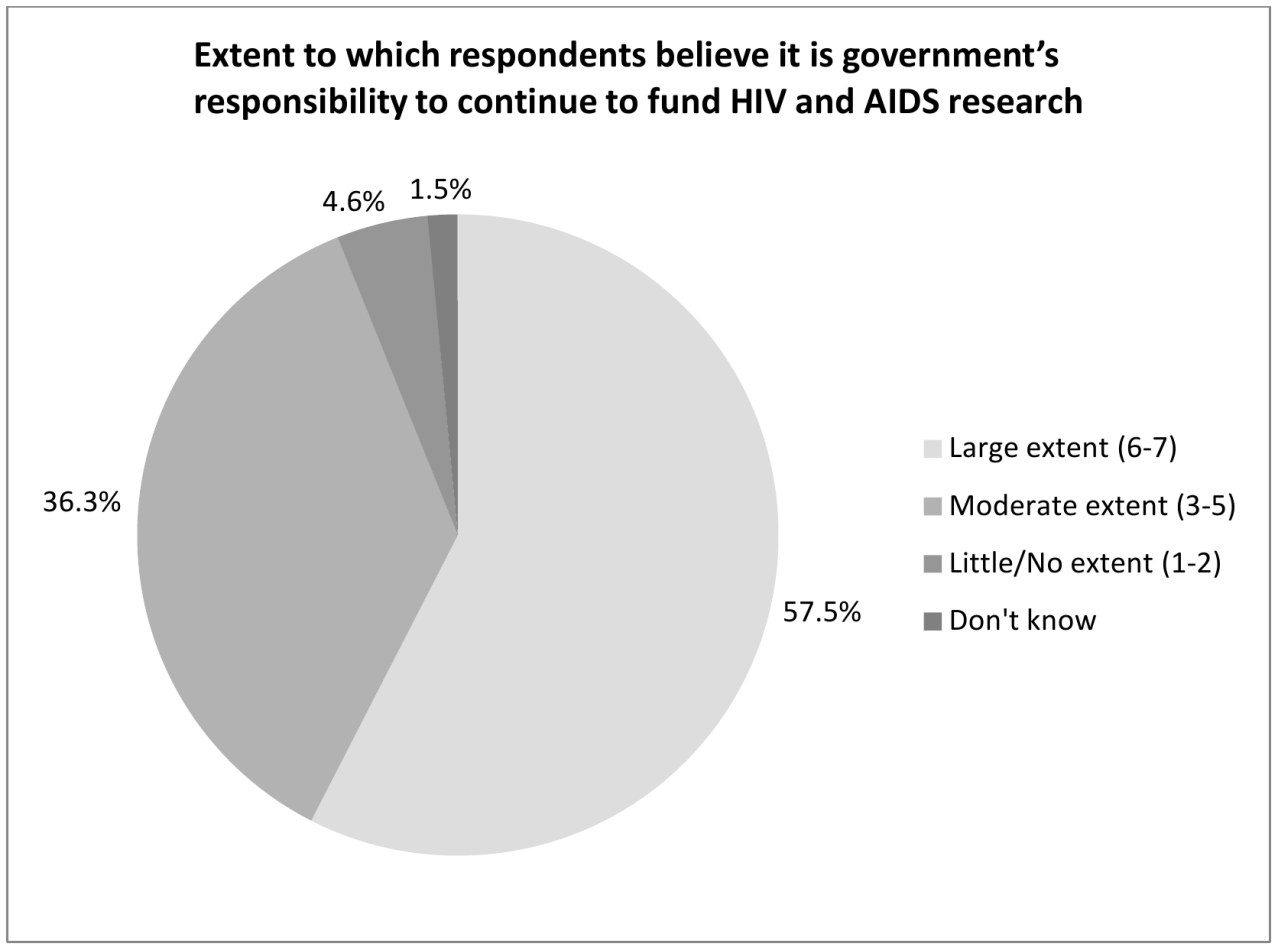
Large extent (6–7). Moderate extent (3–5). Little/No extent (1–2). Don't know.

**Figure 2:**
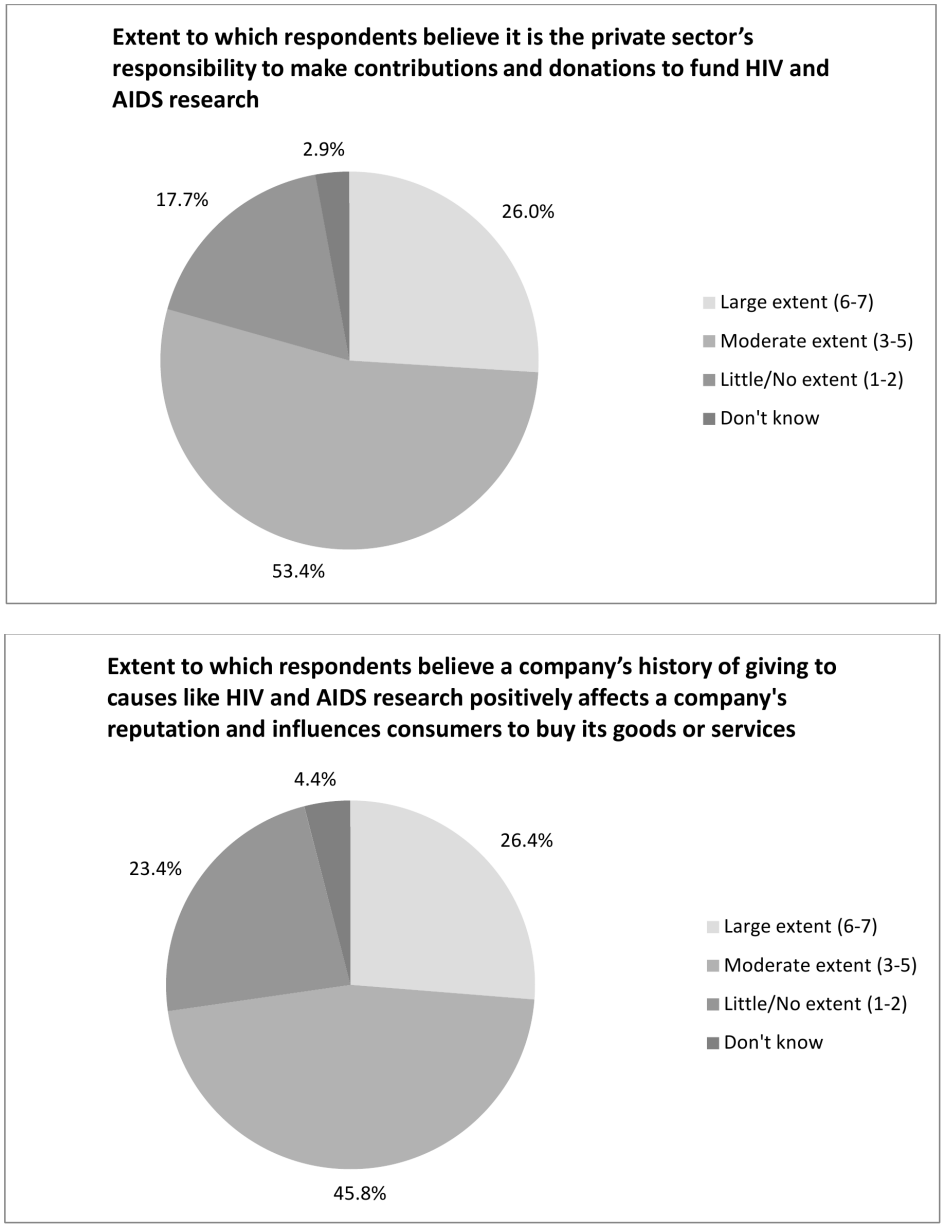
Large extent (6–7). Moderate extent (3–5). Little/No extent (1–2). Don't know.

Overall, survey respondents believed it was government's responsibility to continue to fund HIV and AIDS research (58% believed this to a large extent and 36% believed this to a moderate extent) (Figure 1). A majority also believed that the private sector had a responsibility to make contributions and donations to fund HIV and AIDS research (26% to a large extent and 53% to a moderate extent) (Figure 2). About one quarter (27%) indicated that a company's history of giving to causes like HIV and AIDS research positively affects their willingness to be a consumer of that company.

## Discussion

This analysis reveals that Canadians are a charitable people, with a majority of survey respondents having donated to a charitable cause in the past 12 months. Donations were directed primarily to charities seeking treatment or a cure for diseases and illnesses, although significant proportions of Canadians reported donations to charities assisting children, to disaster relief agencies, to charities dealing with poverty and homelessness and to hospitals. Smaller proportions of this donor group reported donations to charities that addressed international development, the environment and universities or other educational institutions.

Cancer charities were, by far, the most frequent beneficiaries of respondents' donations from those who donated to illness or disease charities. Indeed, almost all respondents reported that they had ever made a donation to a cancer organization. Just under one-in-ten of those who had made a donation to an organization focused on a disease or illness reported donations to organizations focused specifically on HIV and AIDS in the past 12 months, with just over one-in-five ever having made a donation to HIV and AIDS.

### Relationship between Proximity to an Issue and Subsequent Philanthropy

In a cross-national comparison of national campaigns for charitable causes in the Netherlands, Spain, Sweden, and the United States undertaken between 1950 and 2011, van Leeuwen and Wiepking (2012) compared potential determinants of success, namely perceived characteristics of recipients, characteristics of the donor, and structural characteristics of national campaigns [8]. The authors noted a relationship in the proximity between donor and recipient, suggesting that geographical or cultural proximity can translate to increased generosity. This is in concordance with the work of Small and Simonsohn (2008), who in exploring reasons why people give to different causes found that proximity in the form of sympathy, empathy or association to a cause, malady or to an afflicted person could translate to prosocial behaviour such as increased charitable behaviour toward the focal point of their sympathy or empathy [18]. Merchant, Ford and Rose (2011) integrated lessons learned from a literature review and a series of focus groups to develop a model subsequently tested in several studies [19]. Applying structural equation modelling, these authors concluded that nostalgia on the part of a donor can translate to degrees of emotional and familial utility, which can mediate donor's commitment and hence translate to increased intention to donate. Breeze (2013), in a qualitative study of committed donors, found a tendency for donors to direct their charitable activities to organizations or causes they felt affinity with, could identify with and that related to their own experiences [20].

In our study, while reports of donations specifically to HIV and AIDS were relatively few, almost half of those who had never made a donation to HIV and AIDS indicated a willingness to do so in the future, although those who indicated they were “very likely” to consider making such a donation were a small minority. Nevertheless, the findings reflect an important potential opportunity to broaden charitable giving to HIV and AIDS through the identification and recruitment of donors from the substantial pool of those who would consider giving to HIV and AIDS.

The findings suggest the study population strongly believed that governments have a responsibility to fund HIV and AIDS research. In addition, there was a belief that the private sector has a role to play in donating to HIV and AIDS also. These findings reflect a public expectation that governments continue to fund HIV and AIDS activities, supplemented by the private sector, and by individuals.

The literature on charitable giving highlights, how, across North America, a key concern for non-profit organizations raising charitable donations is to determine ways to enhance empathy in segmented groups of potential donors and appeal to each donor group on their level [21]–[30]. With regard to HIV and AIDS, these target groups may be family of PLHIV, parents in general, young professionals, or those who are members of communities disproportionately affected by HIV [10]. Previous research suggests it would be beneficial for non-profit organizations to learn how to identify and target groups of individuals that are more compassionate and empathic in general as it is probable that these people are already donating in some form, or are active in certain organizations [10].

In order to maintain and increase charitable giving for HIV and AIDS, it will be important for Public Health, and agencies serving it, to provide accurate and reliable information regarding HIV and AIDS as the available evidence indicates that more accurate knowledge on HIV and AIDS is associated with more empathetic and altruistic attitudes toward PLHIV, and by extension may be more associated with overall charitable giving to causes related to HIV and AIDS.

## Limitations

While the findings reported in this paper help shed light on the charitable giving of people in Canada towards HIV and AIDS, they do so under several caveats. First, there exists a well-documented sampling bias between surveys that ask about charitable giving and survey response [31]–[34]. In this survey, efforts were made to address some of the variance in sample response through limited weighting, and while this went some distance to better reflect the Canadian population proportionately, it ultimately would have little effect on nonresponse bias. While there are no perfect statistics, there is also no perfect correction for nonresponse bias. This is an existing if not necessary limitation of the data set. No information is captured about those that chose not to respond. As indicated in Table 1, the weighted sample utilized in this paper's analysis is generally comparable across a set of demographic variables (age, gender and province/territory of residence) identified as important to control for by Public Works and Government Services Canada [14]. However, other aspects of our sample are less representative of the Canadian population, such as education and household income. Study participants were more educated and wealthier than the population of Canada. The median Canadian household income in 2010 was approximately $60,000 before taxes [35]; our study population is skewed toward higher household income with almost 40% of our sample reporting an annual household income of greater than $80,000 before taxes. Further, approximately 53% of Canadians over 15 years of age have completed education after high school in 2011 [36], whereas 77% of our study population reported completing education after high school. While these are important considerations, in the multivariate analysis neither education nor household income were significantly associated with ever donating to HIV and AIDS, which suggests that these population differences can be expected to have a minimal effect on the interpretation of our analyses. That said, we do not have information on the types of people who chose not to participate in our study, and this may contribute to a form of non-response bias we are unable to identify.

In an analysis of the impact of data collection method [37], Public Works and Government Services Canada reported, of studies with stated response rates conducted by the Government of Canada between 2009 and 2011, 88 telephone studies reported an average response rate of 14%, 22 online studies reported an average response rate of 28%, and 14 mixed methods studies reported an average response rate of 21%. A previous national survey of HIV and AIDS in Canada reported that 20 to 25 per cent was a typical rate of response for a national public opinion survey exploring substantive areas like HIV and AIDS using questionnaires of a similar length to ours [5]. The response rate of 25% for the current study is therefore consistent with that of similar surveys. Increasingly, expected rates of response to telephone and online public opinion research in Canada have been in decline, due in part to an overall increase in public opinion research [38]–[39], response fatigue [40]–[41], and evolutions in communication technologies [42]–[43].

Boyle, Lewis and Tefft (2009) reflect how the increasing popularity of cell phone only or cell phone mainly households can impact the coverage bias in landline-oriented survey research [44]. They suggest this is one of the elements influencing the applicability of dual frame surveys, particularly when key population characteristics risk being under (or over) represented in the landline only sampling frames. Voigt, Schwartz, Doody, Lee and Li (2010) concur that the utility of landline only studies need to be considered carefully, owing to the increase in cell phone only or cell phone mainly households [45]. Future approaches to consider would include matched or stratified analyses of the landline/cell phone status of individuals and households, given that in some survey contexts the distributions of demographic characteristics between cell phone, cell phone only or cell phone mainly households may differ substantially from landline households [46].

While the individuals who were recruited to participate in our study were located within Canada, the questions upon which the analysis in this paper is based did not explicitly indicate that only charitable donations to causes in Canada were to be included. Therefore, we cannot be sure what proportion of our respondents donated to causes in Canada versus causes outside of Canada. While questions posed to respondents indicated the type of charities individuals had donated to, questions were not included that sought information on specific charities. Future research would be advised to explore further the nature of the causes that people in Canada donate to, including the internationalization of philanthropy, and patterns of donating to specific causes or charities particularly as they relate to charitable activities over an individual's life course.

The findings reported here are based on cross-sectional survey research, which carries with it a number of limitations [47]–[50]. It can be argued that cross-sectional research is one-dimensional in that it occurs at one point in time. Were this survey to be completed at regular intervals or were similar questions explored longitudinally using an alternate research method, other results may have been found. Given that the research is cross-sectional, inferring causal relationships can be challenging. The analysis therefore identifies factors associated with charitable giving only.
